# How can adherence to guidelines be improved? The case of fibrinolytics

**DOI:** 10.1590/S1516-31802001000600002

**Published:** 2001-11-01

## The importance of national data

There is no doubt that the first step in developing public health policies is to have reliable national data. The paper by Escosteguy et al.^1^ embodies this premise, giving us information about acute myocardial infarction treatment in the city of Rio de Janeiro.

As its main conclusion, the study demonstrated that, as expected, cost-effective drugs such as aspirin, beta-blockers and fibrinolytics are all underutilized. This commentary will discuss the issue of fibrinolytics in more detail: it was shown that almost one third of the patients with indication for the utilization of the drug, and without contraindication, did not use it.

## The guidelines and fibrinolytics utilization in acute myocardial infarction

Great efforts towards improving fibrinolytics utilization among patients with acute myocardial infarction have been made by cardiology societies.^2-3^ Despite this, it seems that improvements have been slow in coming about. The use of streptokinase started in Brazil at the beginning of the 1980s, but increased significantly from 1985 onwards, when IV utilization and the commercialization of the drug took place simultaneously.^4^ Taking into account the number of vials made available for sale, it has been demonstrated that the utilization of fibrinolytic drugs increased for some time. However, it seems that, at least in Rio de Janeiro, this is not the case nowadays.

Why are people not improving the utilization of fibrinolytics among patients without contraindication? Interestingly, we can find some of the answers in another survey done in Rio de Janeiro.^5^ In this paper, it was demonstrated that 17.4% of the emergency units contacted did not have the drug (33.4% in public units). Also, it was found that the chance of reperfusion therapy being administered in the emergency department was low in more than half of the centers contacted.

## The problem of the delay in the utilization of the drug

Escosteguy et al.^1^ found a significant correlation between CCU/ICU admission and thrombolytic drug utilization. On the other hand, Brasileiro et al.^5^ showed that, in the same city, 30.5% of the centers had a policy of beginning utilization of the drug in the CCU/ICU, which certainly leads to an unacceptable delay.

So, it is clear that we have two important problems to be solved. First, a great proportion of patients with indication for thrombolytic drugs, and without contraindication, does not receive the drug. Second, probably most of those submitted to this kind of treatment do not receive it in an appropriate way.

## Is there a solution?

We are convinced that the long-term solution needs to take into account the recommendations described in the guidelines. These recommendations need to be followed by our authorities, who will give support as the public health provider; by our private health insurance companies; and, probably most importantly, by doctors in general. This does not only involve cardiologists, but other specialists as well, like emergency-service and intensive-care professionals. In order to achieve the goal of spreading the guideline recommendations to everybody related to the field, cardiology societies depend, to a large extent, on the support of private and governmental funding. This means that, without the engagement of civil society as a whole, it will be very difficult to reach a satisfactory solution.
